# Independent Replication on Genome-Wide Association Study Signals Identifies *IRF3* as a Novel Locus for Systemic Lupus Erythematosus

**DOI:** 10.3389/fgene.2020.00600

**Published:** 2020-07-03

**Authors:** Feixia Zhang, Yong-Fei Wang, Yan Zhang, Zhiming Lin, Yujie Cao, Huoru Zhang, Zhong-Yi Liu, David L. Morris, Yujun Sheng, Yong Cui, Xuejun Zhang, Timothy J. Vyse, Yu Lung Lau, Wanling Yang, Yanhui Chen

**Affiliations:** ^1^Department of Pediatrics, Union Hospital Affiliated to Fujian Medical University, Fuzhou, China; ^2^Department of Paediatrics and Adolescent Medicine, The University of Hong Kong, Hong Kong, Hong Kong; ^3^Shenzhen Futian Hospital for Rheumatic Disease, Shenzhen, China; ^4^Department of Pediatric Surgery, Guangzhou Women and Children’s Medical Center, Guangzhou, China; ^5^Department of Rheumatology, Third Affiliated Hospital of Sun Yat-sen University, Guangzhou, China; ^6^Division of Genetics and Molecular Medicine, King’s College London, London, United Kingdom; ^7^Department of Dermatology, No.1 Hospital Affiliated to Anhui Medical University, Hefei, China

**Keywords:** genome-wide association study, replication, *IRF3*, systemic lupus erythematosus, lupus nephritis

## Abstract

Systemic lupus erythematosus (SLE) is a genetically complex autoimmune disease. Despite the significant progress made in identifying susceptibility genes for SLE, the genetic architecture of the disease is far from being understood. In this study, we set to replicate a number of suggestive association signals found in genome-wide association studies (GWASs) in additional independent cohorts. Replication studies were performed on Han Chinese cohorts from Hong Kong and Anhui, involving a total of 2,269 cases and 5,073 controls. We identified a missense variant in *IRF3* (rs7251) reaching genome-wide significance through a joint analysis of GWAS and replication data (OR = 0.876, P = 4.40E-08). A significant correlation was observed between rs7251 and lupus nephritis (LN) by subphenotype stratification (OR = 0.785, P = 0.0128). IRF3 is a key molecule in type I interferon production upon nucleic acid antigen stimulations and may inhibit regulatory T cell differentiation. Further elucidation of the mechanism of this association could help us better understand the pathogenesis of SLE.

## Introduction

Systemic lupus erythematosus (SLE) is a prototype autoimmune disease with multi-organ damage. Lupus nephritis (LN), affecting 40–70% of SLE patients, is one of the most severe manifestations and the most common factors causing death (Mohan and Putterman, 2015). There are significant population differences for the disease: overall Africans and Asians have higher disease prevalence, more severe symptoms, and higher morbidity of LN than Europeans do (Danchenko et al., 2006).

Genetic factors play a pivotal role in SLE pathogenesis (Deafen et al., 1992), supported by findings that the risk of developing SLE for the siblings of affected individuals is 30 times higher than that for the general population (Sestak et al., 1999). Environmental factors may serve as triggers for the disease, such as infections by viruses and bacteria, exposure to ultraviolet light, especially for individuals with genetic susceptibility (Doria et al., 2009). Although more than 90 susceptibility loci (Chen et al., 2017) have been identified to be associated with SLE by genome-wide association studies (GWASs) and subsequent replications, the genetic basis of the disease is far from being fully understood, indicating that there are many more additional loci to be discovered.

In this study, we first analyzed the data from two previously published SLE GWAS (Bentham et al., 2015; Morris et al., 2016; Sun et al., 2016). A number of SNPs with suggestive significance were further replicated in three independent cohorts collected from Hong Kong and Anhui, China. A genetic variant (rs7251) located in *IRF3* was identified as being associated with SLE, and further subphenotype analysis found that the SNP had a significant association with LN. Functional annotation of the susceptibility gene also supported the potential pathogenic role of the genetic variant in the disease.

## Methods

### Subjects

The GWAS datasets used in the discovery stage are from published studies (Bentham et al., 2015; Morris et al., 2016; Sun et al., 2016), with European cohorts comprising 4,943 SLE cases and 8,483 controls (EUR), and Asian cohorts including 2,485 cases and 3,947 controls (AS). The samples included in the replication stage in this study partially overlapped with those used in our previous studies (Yang et al., 2009, 2011, 2013; Wanling et al., 2010; Li et al., 2012; Zhang et al., 2015a,b,c, 2016; Wang et al., 2018), which were collected from Hong Kong (1,255 SLE cases and 951 healthy controls, HK_rep) and Anhui Province, China (1,014 cases and 4,122 controls, AH_rep), respectively.

Briefly, SLE cases in the Hong Kong cohort were recruited from five public hospitals in Hong Kong, namely Queen Mary Hospital, Tuen Mun Hospital, Queen Elizabeth Hospital, Princess Margaret Hospital, and Pamela Youde Nethersole Eastern Hospital. Corresponding controls in the Hong Kong cohort were healthy blood donors at the Hong Kong Red Cross, who were all of self-reported Chinese ethnicity and living in Hong Kong (Yang et al., 2009; Wanling et al., 2010). Detailed clinical records for 1,069 SLE cases in the Hong Kong cohort were available for subphenotype stratification. Cases in the Anhui cohort were collected from several hospitals in central and southern China, and the corresponding controls were ethnically and geographically matched with the cases. Clinical evaluations were performed to exclude any autoimmune disorders in the controls or family history of autoimmune disease (Yang et al., 2009; Wanling et al., 2010). All the cases fulfilled the revised criteria of the American College of Rheumatology for diagnosis of SLE (Hochberg, 1997). All studies were approved by the corresponding institutional review boards mentioned above, and all subjects provided informed consent.

### Candidate Loci Selection in the Discovery Stage

For each GWAS dataset, we conducted imputation using haplotype data from the 1000 Genomes Project by IMPUTE2 (Howie et al., 2012) to infer the genotypes of genetic variants not genotyped or having missed quality control. Single nucleotide polymorphisms (SNPs) with an imputation INFO score <0.9 were filtered out. We also removed SNPs with a genotype call rate <90% or minor allele frequency <1%, as well as subjects with >5% missing data. Hardy–Weinberg equilibrium (HWE) was tested in each GWAS dataset on the controls and SNPs with HWE P < 1.00E-04 were removed. We used PLINK 1.9 for association analysis for data from each cohort, and used METAL (Willer et al., 2010) to perform a meta-analysis to combine association results from different cohorts. SNPs that have a P_*meta*_ > 5.00E-04 or are close to any reported susceptibility SNP for SLE (±200 kbp of the top SNP in a known associated locus) were excluded. After the above analysis, three SNPs with suggestive association signals, rs3008 and rs4763630 and rs7251 were selected for further validation.

### Genotyping in the Replication Stage

SNP rs3008, rs4763630, and rs7251 were genotyped by TaqMan assay (Applied Biosystems, United States, catalog nos. C_2677324_10 for rs3008, C_11360932_10 for rs4763630, and C_7798230_20 for rs7251) in a portion of samples from the HK cohort that was not included in the GWAS stage (753 cases, 768 controls, HK_rep_1). SNP rs7251 was further examined in the remainder of the HK cohort (502 cases, 183 controls, HK_rep_2) and replication cohorts from Anhui, namely the AH_rep. SNP rs3008 and rs4763630 did not show any association signal in the HK_rep_1 cohort and were not further tested for the rest of the study.

### Subphenotype Stratification Analysis

In this study, detailed clinical data on 1,069 cases from the HK cohort was used and 20 clinical symptoms or laboratory test results were analyzed for subphenotype stratification. They include disease onset age, malar rash, discoid rash, photosensitivity, oral ulcer, arthritis, serositis, neurological disorder, hematologic disorder, renal disorder, C3 low, C4 low, ANA, anti-dsDNA, IgG ACA, IgM ACA, anti-Sm, anti-Ro (SSA), anti-La (SSB), and anti-RNP. The association of rs7251 with subphenotypes was analyzed by comparing cases positive for a certain subphenotype with cases negative for that subphenotype, cases positive for a certain subphenotype with controls, and cases negative for a certain subphenotype with controls, using P ≤ 0.05 as the threshold for statistical significance.

## Results

### Identification of rs7251 as a Novel SLE Susceptibility Locus

In the discovery stage, SNP rs3008, rs4763630, and rs7251, with suggestive association from SLE GWAS datasets, were chosen for further validation. The three SNPs were first genotyped in the HK_rep_1 cohort (753 cases and 768 controls) by TaqMan methods, and rs7251 was further examined in the HK_rep_2 and AH_rep cohorts using the same method, while the other two SNPs were not further explored (see Supplementary Table S1). The replication study included a total of 2,269 SLE cases and 5,073 healthy controls of all self-declared Chinese ancestry. A meta-analysis was performed based on the results from both GWAS and replication stages, altogether including a total of 7,212 cases and 13,556 controls. The result for rs7251 is shown in Table 1.

**TABLE 1:** Summary statistics for rs7251 in discovery stage.

SNP	Gene	Position	Chr	Alleles	Cohorts (cases/controls)	F_A	F_U	P	OR	P_het
rs7251	*IRF3*	50162909	19	C/G	Meta (7212/13556)	–	–	4.40E-08	0.876	0.767
					EUR GWAS (4943/8483)	–	–	1.79E-05	0.880	
					HK_rep (1255/951)	0.313	0.337	0.102	0.898	
					AH_rep (1014/4122)	0.334	0.372	0.002	0.848	

### Association of *IRF3* rs7251 With SLE Subphenotypes

Making use of the available clinical data for 1,069 SLE cases in the HK cohort, we performed a subphenotype stratification analysis for rs7251 (Table 2). It was found that rs7251 was significantly associated with LN when comparing cases positive on LN with those who are negative (OR = 0.785, P = 0.0128), while the SNP did not show evidence of association for the cases negative on LN when compared with the controls (OR = 0.990, P = 0.895). In addition, antinuclear antibodies (ANA) and anti-ds-DNA antibodies showed a trend of association with the SNP but did not reach statistical significance (Table 2).

**TABLE 2:** Association of *IRF3* rs7251 with SLE analyzed by subphenotype stratification.

Subphenotype	case(+)	case(−)	Control	Case (+) vs case (−)		Case (+) vs control		Case (−) vs control	
	n	n	n	OR	P	OR	P	OR	P
Onset age	258	685	951	0.959	0.719	0.832	0.0944	0.867	0.0657
Malar rash	613	456	951	0.979	0.823	0.886	0.131	0.906	0.257
Discoid rash	89	980	951	1.3	0.109	1.14	0.43	0.874	0.054
Photosensitivity	275	794	951	1.04	0.695	0.924	0.457	0.885	0.0972
Oral ulcer	233	836	951	1.05	0.671	0.93	0.52	0.885	0.0935
Arthritis	588	481	951	0.978	0.813	0.886	0.133	0.906	0.251
Serositis	150	919	951	0.969	0.82	0.871	0.314	0.899	0.131
Neurological disorder	72	997	951	1.11	0.587	0.986	0.941	0.889	0.0886
Hematologic disorder	632	437	951	1.08	0.406	0.925	0.319	0.853	0.0753
**Renal disorder**	**449**	**620**	**951**	**0.785**	**0.0128**	**0.777**	**0.00471**	**0.99**	**0.895**
C3 Low	599	470	951	1.02	0.835	0.903	0.202	0.885	0.158
C4 Low	205	864	951	1.24	0.0699	1.06	0.595	0.858	0.0337
**ANA**	852	217	951	0.819	0.0855	**0.858**	**0.0349**	1.05	0.685
Anti-dsDNA	764	305	951	0.93	0.492	0.877	0.077	0.943	0.561
IgG ACA	251	818	951	1.16	0.191	0.998	0.985	0.864	0.0463
IgM ACA	158	911	951	1.19	0.186	1.03	0.796	0.871	0.0525
Anti-Sm	110	959	951	1.16	0.333	1.02	0.892	0.881	0.0702
Anti-Ro(SSA)	484	585	951	1.15	0.135	0.967	0.692	0.838	0.0298
Anti-La(SSB)	122	947	951	1.49	0.00516	1.27	0.0914	0.853	0.0239
Anti-RNP	212	857	951	1.34	0.0109	1.13	0.285	0.841	0.0173

*case(+), SLE patients positive for a certain phenotype. case(−), SLE patients negative for a certain phenotype. Onset age, cases with the age of onset = 20 years were regarded as case(+), the age of onset >20 years regarded as case(−). rs7251 was significantly associated with LN when comparing cases positive on LN with those who are negative (OR = 0.785, P = 0.0128), while the SNP did not show association relationship between case(−) and control (OR = 0.99, P = 0.895).*

### Functional Annotation for *IRF3* rs7251

SNP rs7251 is located in exon 10 of *IRF3*, resulting in a threonine to serine substitution in amino acid position 427, the last amino acid of the protein (T427S). The risk allele G encodes for serine, which is a major allele in most ethnicities except in Africans, for whom the C allele encoding for Threonine is the major allele. It seems that the C allele is the ancestral allele and the risk G allele is the derived allele (Supplementary Figure S1). The C terminus of IRF3 is well conserved, although the last few amino acids are only conserved in primates. Whether the SNP is causal, and if it is, whether the amino acid substitution is the mechanism, requires further studies.

On the other hand, blood eQTL data shows that rs7251 is a cis-eQTL for *IRF3* (P = 1.87E-109)^1^. Information from GTEx also shows that rs7251 is highly associated with expression levels of *IRF3* in various tissues, such as spleen, cervix, thyroid, etc.^2^, and the risk allele (rs7251-G) is associated with increased expression of *IRF3*^3^. Thus, it is likely that rs7251 associates with SLE through an increasing expression of *IRF3*.

## Discussion

In this study, we identified a novel susceptibility variant (rs7251 in *IRF3*) associated with SLE by replication of GWAS signals in additional independent cohorts, altogether involving a total of 7,212 cases and 13,556 corresponding controls of both European and East Asian (Chinese) descent. By analyzing the correlation between the genotypes and subphenotypes, we also found that the risk locus is likely involved in LN.

Several members of the IRF gene family have been found associated with SLE, including IRF5 (Bentham et al., 2015; Morris et al., 2016), IRF7 (Morris et al., 2016; Langefeld et al., 2017), and IRF8 (Chen et al., 2017). However, the role of IRF3 in SLE susceptibility was unclear. A previous study suggested that rs2304206 in *IRF3* (which has intermediate LD with rs7251: *R*^2^ = 0.68 in Europeans, *R*^2^ = 0.43 in East Asians between the two SNPs) could be associated with SLE, based on a cohort of Mexican Mestizo descent with 156 SLE patients and 272 controls (Santana-de Anda et al., 2014). However, another study failed to confirm the genetic association of *IRF3* (rs7251, rs2304204, and rs2304207) with SLE in a Spanish population comprised of 610 SLE patients and 730 healthy controls (Sanchez et al., 2009). Our study is the first one to confirm the association of IRF3 with the disease (OR = 0.876, P = 4.40E-08), based on a large sample size from multiple cohorts of two major ethnicities.

**FIGURE 1:**
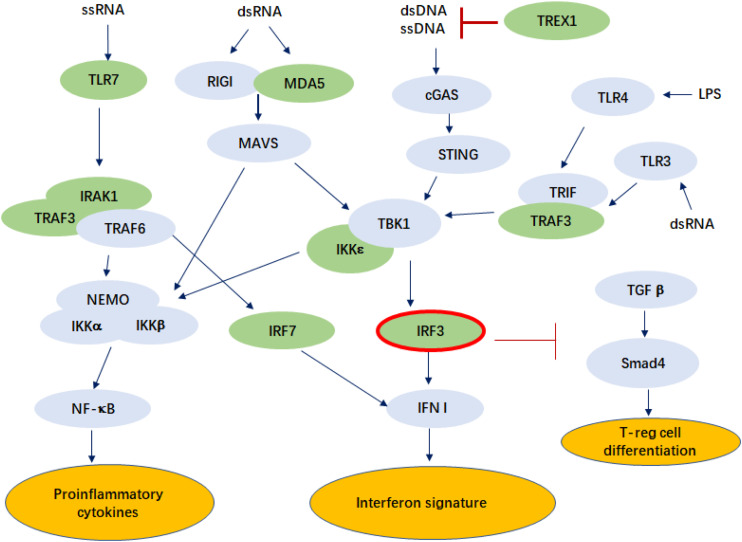
Pathways in which IRF3 is involved that may play a role in the pathogenesis of SLE. The genes known to be associated with SLE are colored in green. ssRNA, single-stranded ribonucleic acid; dsDNA, double-stranded ribonucleic acid; TLR, Toll-like receptor; IRAK1, interleukin-1 receptor associated kinase 1; TRAF3, tumor necrosis factor receptor associated factor 3; NEMO, nuclear factor-kappa B essential modulator; IKKα, IκB Kinase α; RIGI, retinoic acid-inducible gene I; MDA5, melanoma differentiation-associated protein 5; MAVS, mitochondrial antiviral signaling protein; cGAS, cyclic GMP-AMP Synthase; STING, stimulator of interferon genes; TBK1, TANK-binding kinase 1; IRF, interferon regulatory factor; IFN I, type I interferon; TREX1, three prime repair exonuclease 1; TRIF, Toll–IL-1 receptor domain-containing adaptor inducing IFN-β; TGFβ, transforming growth factor β; Smad4, mothers against decapentaplegic homolog 4; LPS, lipopolysaccharide.

Our study also found that *IRF3* rs7251 is particularly associated with LN, a major morbidity in SLE that often leads to end-stage organ failure (Table 2). It has been recognized that the TLR3 signaling contributes to the pathogenesis of LN (Lorenz et al., 2017; Devarapu and Anders, 2018). Another study found that repeated stimulation by polyI:C injected to lupus-prone MRL^*lpr/lpr*^ mice aggravated nephritis by chronic TLR3 activation on glomerular mesangial cells and antigen-presenting cells (Patole et al., 2005). *IRF3* is a critical element downstream of TLR3 activation and a key molecule in type I interferon production (Figure 1).

SNP rs7251 is a missense variant of *IRF3* that changes serine to threonine on the last amino acid of IRF3 (T427S). Serine is a derived allele that might have a different function than Threonine encoded by the ancestral allele. On the other hand, blood eQTL and GTEx data clearly show that rs7251 is a cis-eOTL for *IRF3*, and the SLE risk allele (rs7251-G) is associated with increased expression of *IRF3* in all tissues and organs examined in GTEx^4^. The DICE data [(Database of Immune Cell Expression), Expression quantitative trait loci (eQTLs) and Epigenomics] indicates that *IRF3* is highly expressed in immune cells, especially in T cells (Supplementary Figure S2). More studies are needed to elucidate the mechanism of this locus in the pathogenesis of SLE.

Exogenous ligands, produced by infection and endogenous ligands produced by apoptosis, activate TLRs and initiate downstream signaling cascade, resulting in high production of type I IFN and inflammatory cytokines, as well as immune cell activation (Wu et al., 2015; Figure 1). IRF3 is a key molecule in viral infection-induced type I interferon signaling, while type I interferon signaling is a key signature in SLE pathogenesis. Mutations in *IRF3* were reported to cause herpes simplex encephalitis, presumably due to impaired IFN responses to HSV-1 infection (Andersen et al., 2015). A recent study showed that ischemic cell death and uptake of cell debris by macrophages in the heart induce activation of IRF3 and IFNI production, which plays a critical role in myocardial infarction (Kevin et al., 2017). In addition, IRF3 might also be involved in SLE pathogenesis through T cell differentiation. Activated IRF3 was found to play a role in RIG-I-like receptors (RLR) signaling that represses TGF-β responses in innate host defense. This may include repression of the TGF-β-induced generation of T-reg effector lymphocytes, through repression of Smad4 (Xu et al., 2014).

Viral or host dsRNA activates *MDA5*, which is encoded by *IFIH1*, a gene known to be associated with the disease and is highly upregulated in the immune cells of SLE patients (Robinson et al., 2011; Cen et al., 2013). Loss of function mutations in *TREX1* (reference) and gain-of-function mutations of *IFIH1* are both known to cause lupus-like phenotypes in humans and animal models (Rice et al., 2014), while both are involved in the pathways that activate *IRF3* (Figure 1).

We recognize the limitations of the study, especially on subphenotype analysis, which has a small study power and may not be significant when multiple testing burden is taken into consideration. Subphenotype analysis has low power in general and could be affected by bias in ascertainment, and may be different in different ancestral groups. Future independent replications are necessary to confirm these findings. The benefit of understanding the role of genetics in clinical heterogeneity of the disease can only be realized when findings from independent cohorts provide support to each other.

There is a long way to go before genetic findings could lead to clinical applications, as an individual locus, such as *IRF3*, only has small contribution toward disease susceptibility. Clinically relevant stratification of patients for better treatment and prognosis will have to rely on a much better understanding of the genetic architecture of the disease and a comprehensive analysis of genome-wide data. The polygenic risk score approach is starting to paint a future in which genetics plays a role in clinical intervention of complex diseases (Khera et al., 2018). As an extremely heterogeneous disease, SLE could benefit enormously from genetics if we can tell the genetic differences among patients.

## Conclusion

In summary, we identified a genetic variant in *IRF3* (rs7251) associated with SLE. In addition, subphenotype analysis indicated that *IRF3* (rs7251) is particularly associated with LN. *IRF3* may play a key role in the pathogenesis of the disease, resulting in the production of IFN I and proinflammatory cytokines, and repression of regulatory T cell.

## Data Availability Statement

The raw data supporting the conclusions of this article will be made available by the authors, without undue reservation, to any qualified researcher.

## Ethics Statement

The studies involving human participants were reviewed and approved by Institutional Review Board of the University of Hong Kong/Hospital Authority Hong Kong West Cluster (HKU/HA HKW IRB).

## Author Contributions

YHC, WY, Y-FW, and FZ conceived the study. Y-FW and YJC performed genetic data analysis and selected SNPs for further validation. FZ, HZ, and Z-YL performed TaqMan genotyping assays. FZ performed association analysis and meta-analysis and wrote the first draft of the manuscript. YZ, ZL, YC, YS, XZ, YL, and WY provided samples for the Chinese cohorts. TV and DM provided the genetic data for the Europe cohort. WY and YHC revised the manuscript. All authors approved the final version of the manuscript.

## Conflict of Interest

The authors declare that the research was conducted in the absence of any commercial or financial relationships that could be construed as a potential conflict of interest.

## Footnotes

1 https://genenetwork.nl/bloodeqtlbrowser/

2 https://gtexportal.org/home/gene/IRF3

3 https://gtexportal.org/home/snp/rs7251#gtexmenu

4 https://gtexportal.org/home/snp/rs7251#gtexmenu

## Supplementary Material

FIGURE S1 IRF3 alignment on different species.

FIGURE S2 The expression of *IRF3* in immune cells.

TABLE S1 Summary statistics of rs3008, rs4763630, rs7251 in GWAS and HK_rep_1.

## Abbreviations

Chr: chromosome
eQTL: expression quantitative trait loci
GWAS: genome-wide association study
HWE: Hardy–Weinberg equilibrium
IFN: type I interferon
IRF: interferon regulatory factor
LD: linkage disequilibrium
LN: lupus nephritis
OR: odds ratio
SLE: systemic lupus erythematosus
SNP: single nucleotide polymorphism
TLR: toll-like receptor.
