# Supplementary material for: Independent Replication on Genome-Wide Association Study Signals Identifies IRF3 as a Novel Locus for Systemic Lupus Erythematosus
Source: Front Genet. 2020 Jul 3;11:600. doi: 10.3389/fgene.2020.00600 (PMC7348047; doi:10.3389/fgene.2020.00600)
Supplement: FIGURE S1 — IRF3 alignment on different species. [file Data_Sheet_1.PDF]

Supplementary Tables and Figure

Supplementary Table 1 Summary statistics of rs3008, rs4763630, rs7251 in GWAS and HK_rep_1 2

Supplementary Figure 1 IRF3 alignment on different species 3

Supplementary Figure 2 The expression of *IRF3* in immune cells. 4

Supplementary Table 1 Summary statistics of rs3008, rs4763630, rs7251 in GWAS and HK_rep_1

SNP	Gene	Position	Chr	Alleles	Cohorts (cases/controls)	F_A	F_U	P	OR
rs3008	*JAK3*	17937429	19	T/C	Meta (3238/4715)	-	-	1.68E-03	1.11
					AS GWAS (2485/3947)	-	-	3.80E-04	1.14
					HK_rep_1 (753/768)	0.449	0.452	0.884	0.989
rs4763630	*DDX12P*	9741500	12	A/G	Meta (3238/4715)	-	-	2.76E-04	0.887
					AS GWAS (2485/3947)	-	-	4.36E-06	0.845
					HK_rep_1 (753/768)	0.431	0.411	0.266	1.086
rs7251	*IRF3*	50162909	19	C/G	Meta (5696/9251)	-	-	1.20E-05	0.885
					EUR GWAS (4943/8483)	-	-	1.79E-05	0.880
					HK_rep_1 (753/768)	0.301	0.325	0.301	0.922

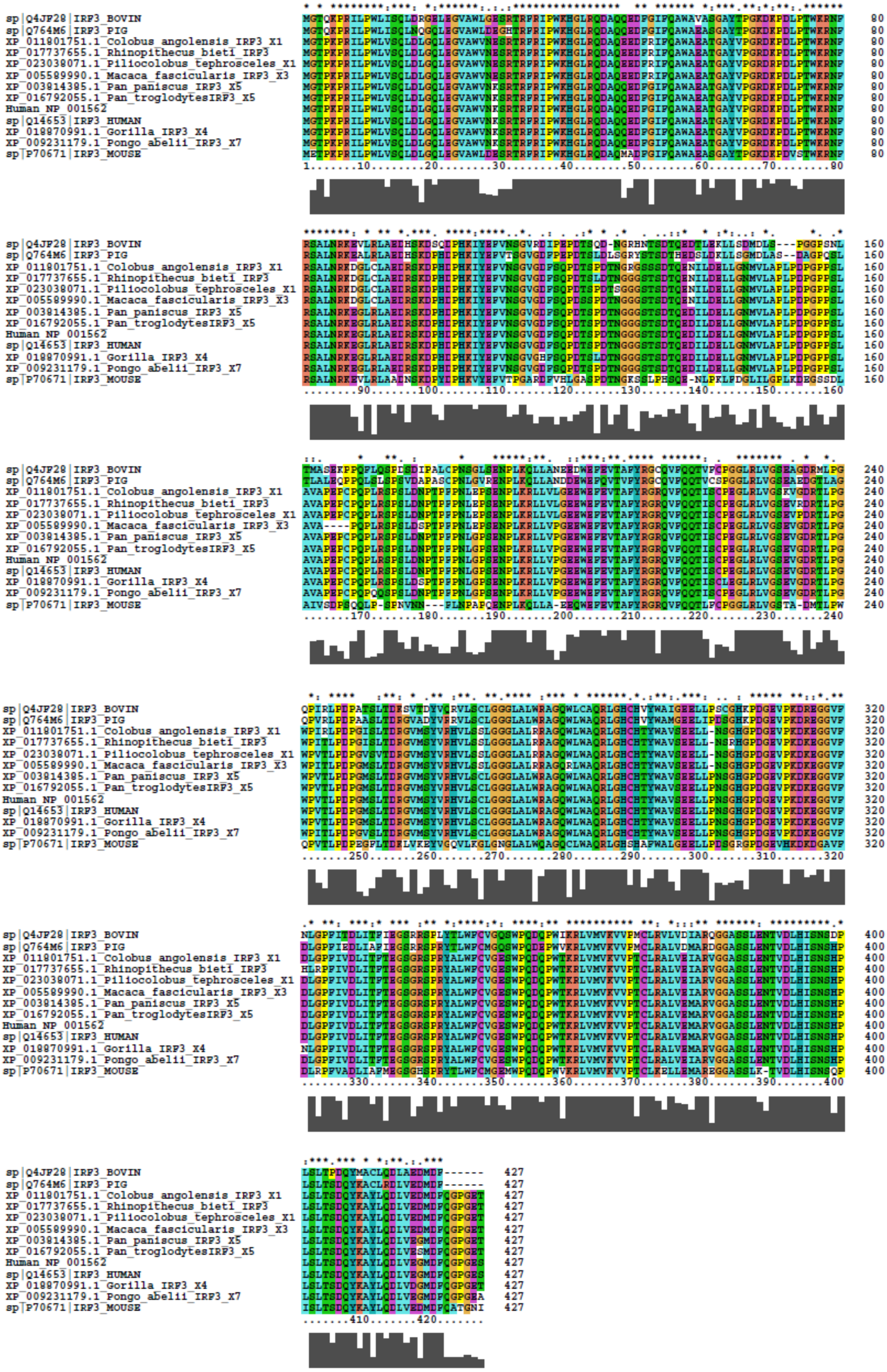

Supplementary Figure 1 IRF3 alignment on different species

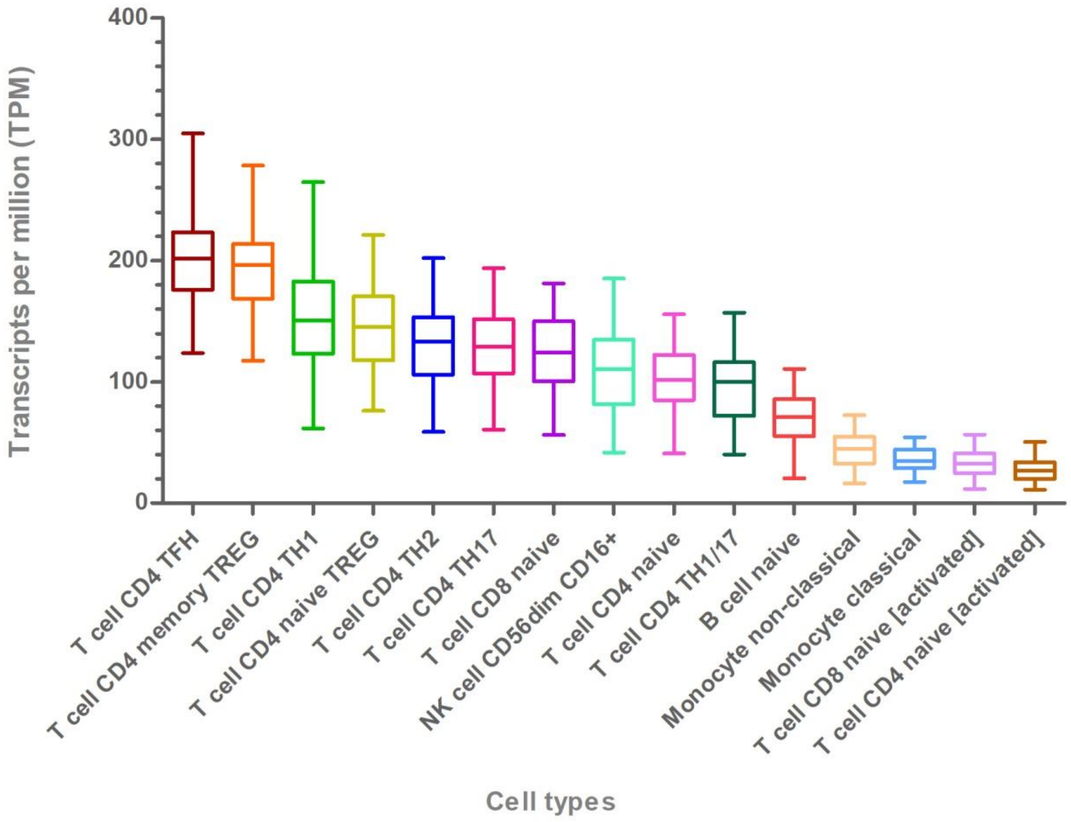

Supplementary Figure 2 The expression of *IRF3* in immune cells.
